# Detection of cannabinoids in hair after cosmetic application of hemp oil

**DOI:** 10.1038/s41598-019-39609-0

**Published:** 2019-02-22

**Authors:** R. Paul, R. Williams, V. Hodson, C. Peake

**Affiliations:** 1 Bournemouth University, Faculty of Science and Technology, Poole, UK; 2 Lextox, The Maltings, Cardiff, UK

## Abstract

The detection of cannabis constituents and metabolites in hair is an established procedure to provide evidence of exposure to cannabis. We present the first known evidence to suggest that applying hemp oil to hair, as cosmetic treatment, may result in the incorporation of Δ^9^-tetrahydrocannabinol (THC), cannabinol (CBN), cannabidiol (CBD) and in one instance, the metabolite 11-hydroxy-Δ^9^-tetrahydrocannabinol (THC-OH). 10 volunteers treated their head hair daily with commercially available hemp oil for a period of 6 weeks. Head hair samples were collected before and after the application period. Hair samples were washed with methanol and subjected to clean up via liquid/liquid and solid phase extraction procedures, and then GC-MS/MS for the analysis of THC, CBN, CBD, THC-OH and THC-COOH. Application of hemp oil to hair resulted in the incorporation of one or more cannabis constituents in 89% of volunteers, and 33% of the group tested positive for the three major constituents, THC, CBN and CBD. One volunteer showed low levels of the metabolite THC-OH. We suggest that cosmetic use of hemp oil should be recorded when sampling head hair for analysis, and that the interpretative value of cannabinoid hair measurements from people reporting application of hemp oil is treated with caution in both criminology and public health.

## Introduction

Cannabis Sativa is a plant species of Cannabis. In addition to its recreational use as a drug of abuse, the plant has widespread alternative uses including the production of food, cosmetics (hemp), textiles and medicinal applications^1^. When toxicology laboratories are required to investigate past exposure to cannabis, analysis of hair can provide powerful evidence. The compounds usually targeted for hair analysis to identify cannabis exposure are: Δ(9)-tetrahydrocannabinol (THC), the main active compound of cannabis, the metabolite [11-nor-Δ(9)-tetrahydrocannabinol-9-carboxylic acid (THC-COOH)] and two cannabinoids (cannabinol (CBN) and cannabidiol (CBD))^2^. Typically passage of these cannabinoids into the hair includes passive diffusion from blood, diffusion from sweat/sebum or external contamination. One of the key questions to be addressed when interpreting the results of cannabinoid hair analysis is that of proof of consumption. Are the results sufficiently clear to suggest cannabis was consumed, or could the results actually be the result of passive exposure to cannabis smoke, or other mechanisms? Passive exposure is defined by an individual being in an environment that is exposed to drugs, an important public health problem. Cannabis smoke can be inhaled or absorbed into the hair by persons other than the intended smoker/user^3^. Researchers have evaluated second-hand cannabis smoke exposure and the corresponding levels of cannabinoids in biological samples^3,4^. Herrmann *et al*. discovered that in unventilated, confined conditions cannabinoid detection was above threshold and higher concentrations of THC and THC-COOH were found predominantly in the blood, urine and hair^4^. THC and THC-COOH have lower incorporation rates in hair in comparison to other bodily matrices. The low presence of THC may be explained by its weak affinity to melanin while the acidic nature of hair may be the reason for the absence of THC-COOH^5^. Along with the levels of cannabis constituents detected in passive exposure, analysis has been conducted to understand what physiological impact exposure has^3^. Past research has shown evidence of increased heart rate and minor impairments in coordination and memory^4,6,7^. Identification of THC/CBN/CBD in hair suggests exposure to cannabis, which could be due to low level or infrequent use of cannabis or historic or passive exposure. However, some argue that the presence of cannabinoids in hair, especially THC is indicative of repeated or chronic exposure^5,8^. The distinction between external contamination and consumption can be difficult for cannabinoid hair analysis^9^, and the implication of a positive test result can have significant consequences for the individual involved. THC-COOH is only formed inside the body, and the presence of this gives unequivocal proof of consumption when detected in hair samples. The metabolite has never been discovered in cannabis smoke ruling out environmental contamination^10^. With hair analysis, THC-COOH is detectable at very low concentrations. The drawbacks for detection from this biological matrix are the requirement for expensive instrumentation and sample preparation can be a more time-consuming process when compared to urine^11^. Routine laboratory screening of hair for cannabis varies and includes the detection of cannabinoids and/or THC-COOH^8^. Hemp is a variety of Cannabis Sativa and is closely related to Cannabis with the difference being in the percentage of THC^12^. Hemp is grown for industrial use and found in food, lotions, medicines, clothing and construction materials. Hemp oil is extracted by pressing the seeds from the female hemp plant^13^. The legalisation of hemp has caused controversy. This is because research has shown that the use or consumption of hemp products could have the potential to impact on drug testing for cannabis^14^.

Hemp oil products are advertised in health shops for their good source of omega fatty acids^15^. Bosy *et al*.^16^ assessed whether oral consumption of hemp oil would negatively affect existing drug screening protocols. Various oils were screened (THC content of bottled oils was 36.0, 117.5, 36.4, 45.7, 21.0, 11.5 mg/g) and administered to volunteers and their urine measured for metabolite levels. GC-MS analysis determined the amount of THC-COOH in each participant’s urine to be below the confirmation cut-off within a 48 hour cessation period. Similarly to hemp oil, hemp foods are classified as ‘natural foods’ and are commercially available. Leson *et al*. showed that daily consumption of hemp food can lead to the presence of THC and THC-COOH in urine, but these compounds were below the confirmation thresholds^17^. These authors^16,17^ suggest that hemp food and oil products do contain cannabinoids but in very low concentrations, and that ingestion of such products should not be deemed as a concern in drug testing. The Cannabis plant has been used in the production of cosmetics through the use of hemp oil and cannabis extracts^18^. An evaluation of Cannabio® shampoo revealed levels of THC, CBD and CBN, three constituents that indicate cannabis exposure^19^. However, normal hygiene practice using the cosmetic produced no positive results in hair. Extreme use could generate positive results for CBN and CBD but not the primary constituent, THC.

Hemp oil is marketed as an effective cosmetic treatment for hair, with claims that direct application of the oil to hair has moisturizing benefits, can aid hair growth, may protect the hair and aid in damage repair, and the oil may add shine to the hair. These claims are unsubstantiated but there is a substantial number of online retailers selling various hemp oil based products intended for direct application to head hair. The composition of these products range from pure hemp oil, to hemp oil included at a relatively low concentration into shampoos and other hair treatments.

In this paper we investigate direct hemp oil application to head hair and the implications on resulting cannabinoid measurements.

## Results

### Cannabinoid concentrations pre and post hemp oil application

Head hair samples were collected from volunteers as described in Methods, and analysed before and after the six week period of hemp oil administration. Results are displayed in Table 1.

**Table 1:** Observed cannabinoid concentrations in hair samples from volunteers.

Volunteer^*^	Before hemp oil application					After hemp oil application				
	THC	CBD	CBN	THC-COOH	THC-OH	THC	CBD	CBN	THC-COOH	THC-OH
1	ND	ND	ND	ND	ND	ND	**4**.**70**	**2**.**80**	ND	ND
2	ND	ND	ND	ND	ND	**0**.**13**	**0**.**03**	**0**.**03**	ND	ND
3	ND	ND	ND	ND	ND	ND	**3**.**26**	**10**.**3**	ND	**0**.**01**
5	ND	ND	ND	ND	ND	ND	ND	ND	ND	ND
6	ND	**0**.**04**	ND	ND	ND	**0**.**04**	**0**.**11**	**0**.**01**	ND	ND
7	ND	ND	ND	ND	ND	ND	ND	**0**.**02**	ND	ND
8	ND	ND	ND	ND	ND	**0**.**02**	**0**.**05**	**0**.**05**	ND	ND
9	ND	ND	ND	ND	ND	ND	**0**.**02**	ND	ND	ND
10	ND	ND	ND	ND	ND	**0**.**01**	ND	ND	ND	ND

All concentrations are from the proximal 0–3 cm section and presented as ng/mg and ND refers to ‘not detected’.

*Volunteer 4 displayed levels of THC, CBD and CBN before hemp oil application, and was therefore removed from any further analysis.

### Hemp oil analysis

The hemp oil product used for the study can be found on the open market. We analysed the oil using gas chromatography coupled to tandem mass spectrometry (GC/MS-MS) and found the following: CBN was detected at 407 ng/ml, CBD, THC, THC-OH and THC-COOH were not detected. The hemp oil did contain an analyte peak in the THC channel, but this peak did not meet peak acceptance criteria. This peak could not be identified.

## Discussion

Despite declaring no consumption or exposure to Cannabis, volunteer 4 showed levels of CBD, THC and CBN prior to applying the hemp oil. For this reason volunteer 4 was excluded from further discussion. Three of the remaining volunteers (volunteers 2, 6 and 8) showed levels of all three major constituents of cannabis (CBD, THC, CBN) in their hair following application of hemp oil. This is an important finding with potential ramifications for cannabinoid hair testing where donors report application of hemp oil. Taylor *et al*.^20^ demonstrated that 77% of self-reported heavy users of cannabis tested positive in hair for THC, 19% for CBD, and 73% for CBN. Of these heavy users the detection of metabolites THC-OH (found in 19% of the sample) and THC-COOH (found in 54% of the samples) were important indicators of consumption. However in the same study, light users of cannabis tested positive for THC (39%), CBN (29%) and CBD (11%), and importantly only 3% and 11% tested positive for metabolites THC-OH and THC-COOH respectively. There may be a risk where laboratories observe detectable hair concentrations of CBD, THC, and CBN in donors who use hemp oil cosmetically on their hair that the result interpretation could mistakenly suggest light cannabis use, or passive exposure. In Table 2 we present our interpretation of volunteer hair cannabinoid concentrations post hemp oil application if we imagine that hemp oil application *was not* declared. If we saw these results with no indication that hemp oil had been applied to the hair, then 6 of the volunteers have cannabinoid levels consistent with low level or infrequent cannabis use, but exposure to cannabis could also explain the results. THC-OH present in volunteer 3 would suggest cannabis use, and the levels of CBN and CBD also present may suggest possible use of a cannabis based product. In a similar manner the CBN and CBD levels in volunteer 1 also suggest possible use of a cannabis based product, but here the absence of THC and any metabolites do not suggest cannabis use.

**Table 2:** Interpretation of volunteer hair cannabinoid concentrations under the assumption that hemp oil administration had not been reported.

Volunteer	Interpretation
1	Not consistent with cannabis use due to the absence of THC and the levels of CBD and CBN present. Likely to be the result of using a cannabis based product. This is not a typical result. Our laboratory has not encountered a result with no THC presence, but high CBD and CBN.
2	Suggestive of low level or infrequent use of cannabis. Could also arise from exposure to cannabis.
3	Suggests cannabis use as the metabolite THC-OH was detected. The level of CBN and CBD present suggest possible use of cannabis based product.
5	No evidence of use
6	Suggestive of low level or infrequent use of cannabis. Could also arise from exposure to cannabis.
7	Suggestive of low level or infrequent use of cannabis. Could also arise from exposure to cannabis. Could also be due to the use of a cannabis based product
8	Suggestive of low level or infrequent use of cannabis. Could also arise from exposure to cannabis.
9	Suggestive of low level or infrequent use of cannabis. Could also arise from exposure to cannabis. Could also be due to the use of a cannabis based product
10	Suggestive of low level or infrequent use of cannabis. Could also arise from exposure to cannabis.

When results post hemp oil application were reviewed it was observed that there was a strong analyte peak present in the THC channel for volunteers 1 and 3 that had a very similar retention time to that of THC at a high level. These concentrations have been reported as not detected as they do not meet the criteria to be reported as THC. Although the hemp oil used in this study did not contain detectable levels of THC, the oil did contain an unidentified peak within the THC channel as described above in the discussion of volunteers 1 and 3. It appears that a structurally similar compound with similar but not exact retention time has transferred during cosmetic application of the oil, and become incorporated in the hair. Analysts should be aware of potential THC analogues in hair where donors report hemp oil application. Routine analytical criteria for peak acceptance should suffice.

During the 6 week hemp oil application period, volunteers followed their usual hair hygiene routine which varied from washing and shampooing daily, to every few days. Oil was applied in the evening, and all volunteers with the exception of volunteers 1 and 3, always washed their hair the following morning. Volunteers 1 and 3 reported some hair washing the morning following oil application, but on occasion hair washing was delayed by 1 to 2 days. Volunteers 1 and 3 display the highest levels of CBN and CBD post hemp oil application. It is possible that the additional time that hemp oil was in contact with the hair prior to hair washing may have caused this increase in concentration. Chromatography from the hair analysis of these same volunteers also showed high levels of the potential THC analogue described earlier, and we suspect the high levels are again a possible result of the less frequent hair washing than other volunteers.

The presence of the metabolite THC-OH in the hair of volunteer 3 is of interest. THC-OH is formed from the metabolism of cannabis, and the compound was not present in the hemp oil itself, or in any other volunteers post hemp oil application. As such it seems unlikely that THC-OH has arisen in this sample as a result of hemp oil application and although volunteer 3 denies consumption or exposure to cannabis during the 6 week study period, we cannot exclude the possibility that this has occurred. Franz *et al*.^21^ observed hair THC-OH concentrations ranging from 0.05–37.6 pg/mg. In our study volunteer 3 testing at 10 pg/mg is consistent with these findings.

Citti *et al*.^22^ investigated cannabinoid concentration in 13 hemp oils, and discovered a wide concentration range of the key cannabinoids across the different oils. In their study cannabis constituents were present in the oils at levels ranging from: THC < LOD to 1.804 ppm (±0.317), CBD < LOD to 1056 (±21.84), CBN < LOQ to 12.41 ppm (±0.850). In our present study all volunteers used the same hemp oil. It is likely therefore that different hemp oils may show higher or lower levels of cannabinoids in hair than our results suggest following hemp oil application.

A possible explanation of the THC levels in hair from volunteers 2, 6, 8 and 10, post hemp oil application, despite the hemp oil testing negative for THC, could be the decarboxylation of any Δ^9^-tetrahydrocannabinolic acid (THCA-A) possibly present in the hemp oil. It has been shown^20^ that some hemp oils contain quite high levels of both THCA-A, and cannabidiolic acid (CBD-A). High CBD hair values in our study post hemp oil application could also have partially been formed in a similar manner, this time via the decarboxylation of CBD-A. Perrotin-Brunel *et al*.^23^ explain that the decarboxylation of THC-A to the psychoactive THC can be induced by light or heat, and the same is true of CBD-A^21^. It could therefore be hypothesized that the hemp oil used in our study may have contained THCA-A and CBD-A (our analytical method does not assess these compounds) which upon frequent application to hair which is subjected to light (daylight) and heat (hair dryer) has undergone decarboxylation to THC and CBD respectively. Both THCA-A and CBD-A have been documented in hemp oils^22^ with levels as high as 9.462 ppm (±0.514) and 821.1 ppm (±13.22) respectively.

It has also been demonstrated^24^ that experimental conditions during the analytical procedure for extraction of cannabinoids from hair can influence the quantitative results. This was demonstrated to be due mainly to the decarboxylation of THCA‐A during procedures involving alkaline hydrolysis and/or elevated temperatures. Such decarboxylation of THCA-A to THC leads to artifactually elevated THC concentrations. In the context of our study, our analytical procedure employed methanolic extraction and a NaOH digestion step. Any THCA-A present on volunteer hair as a result of external contamination could theoretically have been at least partially converted to THC during the analytical procedure. However, our wash procedure has been validated to effectively remove external contaminants so whilst this theory is a viable explanation for any method involving alkaline hydrolysis, it may not be the case for our study.

A possible limitation of our study is the frequency of hemp oil application to hair. During our experiment volunteers applied the oil once daily for 6 weeks. It is likely that in routine use some people may apply hemp oil less frequently to hair, although the duration over which they apply hemp oil prior to testing will of course be highly variable.

THC-COOH remains an invaluable indicator of cannabis use. It is only formed after consumption of cannabis, has not been found in cannabis smoke, and was not found in any of the volunteers from our study following hemp oil application to hair. It is of concern that cannabinoids have been detected in hair samples following the application of hemp oil as a cosmetic procedure, and at levels in our study relevant to suggest cannabis exposure in some cases. By comparison, it has long been established that in cannabis users the cannabis constituent CBD can be present in hair at concentrations between 0.03 and 3.0 ng/mg, CBN at 0.01 to 1.07 ng/mg and THC at 0.1 to 0.29 ng/mg^25^. We suggest that cosmetic use of hemp oil should be recorded when sampling head hair for analysis, and that the interpretative value of cannabinoid hair measurements from people reporting application of hemp oil is treated with caution in both criminology and public health.

## Methods

### Ethical approval

The study protocol was approved by the Ethics Committee of Bournemouth University. All research was performed in accordance with relevant guidelines and regulations. Informed consent was obtained from all participants.

### Analysis of hemp oil

100 µl of Granovita organic hemp oil was transferred into a mixture of 1 ml sodium hydroxide (1 M), 1 g ammonium sulphate and 2.2 ml 9:1 chloroform:propan-2-ol and 50 µl of internal standard (Cannabidiol-D3, THC-D3, Cannabinol-D3, THC-OH-D3, THC-COOH-D3). This was shaken for 20 minutes and centrifuged for 5 mins. To the organic layer 1 ml of drug extraction buffer was added (pH 7.2). Clean up was achieved using Biotage Isolute HCX mixed mode solid phase extraction (SPE). SPE elution was achieved using 80:20 hexane:ethyl acetate. The eluent was dried down at 50 °C for 1 hour and derivatised using 20 µl BSTFA. 2 ul was used for GC-MS/MS analysis.

### Participants

10 volunteers participated in the study, 5 female and 5 male. Volunteer details are presented below in Table 3.

**Table 3:** Age, hair color and gender of volunteers.

Volunteer number	Age	Sex	Hair color
1	22	Female	Brown
2	21	Female	Brown
3	25	Female	Brown
4	21	Female	Brown
5	40	Female	Black
6	32	Male	Black
7	30	Male	Brown
8	41	Male	Brown
9	36	Male	Brown
10	32	Male	Brown

Before participating in this study all volunteers declared that they do not smoke or ingest cannabis in any form, nor are they exposed to cannabis (smoke or otherwise) in any form via passive exposure. Volunteers treated their head daily with commercially available hemp oil during a 6 week period. Volunteers followed their usual hair hygiene routine which varied from washing and shampooing daily, to every few days. Volunteers applied approximately 2 ml of the oil all over their head hair, gently massaging the oil into the hair. Oil was applied in the evening and all volunteers, with the exception of number 1 and 3, always washed their hair the following morning. Volunteers 1 and 3 reported some hair washing the morning following oil application but on occasion hair washing was delayed by 1 to 2 days.

Hair was cut close to the scalp, aiming for the thickness of a pencil. Hair samples were stored in aluminum foil (covered) indicating root end. Hair samples were prepared as a 0–3 cm section from the proximal end. A minimum weight of 10 mg hair was used for analysis.

### Decontamination

The decontamination process aims to remove the external contamination and sebum, hair care products and sweat, whilst minimizing the removal of compounds of interest from within the hair matrix. For this study all hair samples were washed before analysis with methanol.

### Extraction

The extraction protocol is a fully validated method involving overnight extraction with methanol in an ultrasonic bath for a minimum of 4 hours. The methanol is removed and dried down in a centrifugal concentrator. This step is designed to extract the cannabinoids. The hair is then digested in sodium hydroxide and undergoes liquid/liquid extraction using chloroform and propan-2-ol where it is recombined with the concentrated methanol samples. This step is designed to extract other drug compounds that are analyzed in our laboratory. Combined extracts are then dried down fully at 30 °C for 1 hour in a concentrator and, once dry, a buffer solution is added.

### Clean-up by SPE

The samples are taken through a SPE clean-up process using mixed mode cartridges. This is a two-stage process that separates the cannabinoids for analysis by GC-MS/MS and other drugs of abuse for analysis by LC-MS/MS. The eluent containing the cannabinoids is dried down at 50 °C for 1 hour and derivatised using BSTFA for injection onto GC-MS/MS.

### Analysis by GC-MS/MS

GC Method: Oven initial temperature 150 °C. After one minute ramp 50 °C/min for 1 minute, 20 °C for a further 6 minutes to hold at a final temp of 320 °C for 1 minute. Operates in splitless mode with the injector set to 280 °C. The column used was a 15 m × 0.25 mm × 0.25 µm Varian VF-5ms GC column.

Positive Electron Ionization (PEI) carrier gas was helium, collision gas was Argon, and the acquisition mode was multiple reaction monitoring (MRM).

Mass spectrometry multiple reaction monitoring (MS MRM) transitions used are shown in Table 4. Relative standard deviation (% RSD), limit of detection (LOD) and limit of quantitation (LOQ) for the method are detailed in Table 5. LOD and LOQ in Table 5 are relevant to the hemp oil analysis as well as the hair analysis. LOD is defined as the minimum detectable concentration of target compound that provides a signal to noise ratio of at least 3:1. Limit of quantitation is defined as the minimum quantifiable concentration of target compound producing a signal to noise ratio of at least 10:1. Calibrations were linear with r^2^ > 0.99, using a linear, weighted 1/x equation. Calibration concentrations for the hemp oil analysis and the hair sample analysis were the same and were prepared at six concentrations across the following ranges: THC, CBN and CBD prepared between 0.1 ng/ml and 10.0 ng/ml. THC-OH and THC-COOH prepared between 0.01 ng/ml and 0.1 ng/ml.

**Table 4:** MS MRM transitions for the cannabinoids and deuterated standards.

Compound	Target Transition	Qualifier Transition
Cannabidiol	390.3 > 319.2	390.3 > 301.2
Cannabidiol-D3	393.3 > 304.2	
THC	371.3 > 289.2	386.3 > 315.2
THC-D3	389.3 > 374.3	
Cannabinol	367.2 > 310.2	367.2 > 295.1
Cannabinol-d3	370.2 > 310.2	
THC-OH	371.3 > 305.2	371.3 > 289.2
THC-OH-D3	374.3 > 292.2	
THC-COOH	371.3 > 305.2	371.3 > 289.2
THC-COOH-D3	374.3 > 292.2

**Table 5:** % RSD, LOD and LOQ for the detected cannabinoids.

	THC	CBD	CBN	THC-COOH	THC-OH
% RSD	19%	24%	14%	18%	17%
LOD pg/mg (S/N 3:1)	0.12	0.91	0.09	0.07	0.01
LOQ pg/mg (S/N 10:1)	0.41	3.0	0.31	0.24	0.03

## Data Availability

All data generated or analyzed during this study are included in this published article.
